# MiR-21 Induced Angiogenesis through AKT and ERK Activation and HIF-1α Expression

**DOI:** 10.1371/journal.pone.0019139

**Published:** 2011-04-22

**Authors:** Ling-Zhi Liu, Chongyong Li, Qi Chen, Yi Jing, Richard Carpenter, Yue Jiang, Hsiang-Fu Kung, Lihui Lai, Bing-Hua Jiang

**Affiliations:** 1 Lab of Reproductive Medicine, Department of Pathology, Cancer Center, Nanjing Medical University, Nanjing, Jiangsu, China; 2 Department of Pathology, Anatomy and Cell Biology, and Kimmel Cancer Center, Thomas Jefferson University, Philadelphia, Pennsylvania, United States of America; 3 Faculty of Medicine, The Chinese University of Hong Kong, Hong Kong, China; 4 Institute of Molecular and Chemical Biology, East China Normal University, Shanghai, China; University of Barcelona, Spain

## Abstract

MicroRNAs (miRNAs) are endogenous, small noncoding RNAs that play important roles in various cellular functions and tumor development. Recent studies have indicated that miR-21 is one of the important miRNAs associated with tumor growth and metastasis, but the role and molecular mechanism of miR-21 in regulating tumor angiogenesis remain to be elucidated. In this study, miR-21 was overexpressed by transfecting pre-miR-21 into human prostate cancer cells and tumor angiogenesis was assayed using chicken chorioallantoic membrane (CAM). We found that overexpression of miR-21 in DU145 cells increased the expression of HIF-1α and VEGF, and induced tumor angiogenesis. AKT and extracellular regulated kinases (ERK) 1/2 are activated by miR-21. Inhibition of miR-21 by the antigomir blocked this process. Overexpression of the miR-21 target, PTEN, also inhibited tumor angiogenesis by partially inactivating AKT and ERK and decreasing the expression of HIF-1 and VEGF. The AKT and ERK inhibitors, LY294002 and U0126, suppressed HIF-1α and VEGF expression and angiogenesis. Moreover, inhibition of HIF-1α expression alone abolished miR-21-inducing tumor angiogenesis, indicating that HIF-1α is required for miR-21-upregulated angiogenesis. Therefore, we demonstrate that miR-21 induces tumor angiogenesis through targeting PTEN, leading to activate AKT and ERK1/2 signaling pathways, and thereby enhancing HIF-1α and VEGF expression; HIF-1α is a key downstream target of miR-21 in regulating tumor angiogenesis.

## Introduction

MicroRNAs (miRNAs) are a family of small non-coding RNAs with the length of 21- to 25-nucleotides that regulate the expression of various kinds of cellular proteins by targeting their mRNA expression levels. miRNAs act as gene regulators through translational repression or mRNA degradation via binding of miRNAs to target sites in the 3′-untranslated regions (UTR) of protein-coding transcripts. Approximately 500 miRNA genes have been identified in the human genome, which are involved in regulating development, differentiation, apoptosis and proliferation [1], [2]. Among these miRNAs, miR-21 is one of the well characterized miRNAs and overexpressed in various solid tumors including prostate cancers [3]. Recent studies indicated that several tumor suppressors including phosphatase and tensin homolog deleted on chromosome ten (PTEN) [4], tumor suppressor gene tropomyosin 1 [5], programmed cell death 4 [6], [7], maspin [8], and matrix metalloproteinases inhibitors RECK and TIMP3 [9] were targets of miR-21, suggesting that miR-21 is an important oncogenic miRNA which is closely related to tumor growth and metastasis. The expression of miR-21 is also associated with prognosis and the chemosensitivity and therapeutic outcome in colon adenocarcinoma [10], [11]. Moreover, anti-miR-21 suppressed both cell growth of breast cancer *in vitro* and tumor growth in the xenograft model partially through downregulating of the antiapoptotic factor, B-cell lymphoma 2 (Bcl-2) [12]. These data, taken together, support an important role of altered miR-21 expression during tumor development.

Tumor angiogenesis is the process that new vessels sprout from preexisting blood vessels [13]. Angiogenesis is critical for tumor growth and metastasis because tumor will not grow more than 1-2 mm without nutrition and oxygen [14]. Tumor angiogenesis can be triggered by extracellular signals such as growth factors, by genetic alterations such as activation of oncogenes, and by mutations of tumor suppressor genes such as PTEN and p53 [15], [16]. There is limited information about the relationship between miRNA clusters and tumor angiogenesis. Several examples are: miR-17-92 which is upregulated in colonocytes coexpressing K-Ras and c-Myc, represses the expression of anti-angiogenic thrombospondin-1 (Tsp1) and connective tissue growth factor (CTGF), thus induces angiogenesis [17]; miR-378 promotes angiogenesis induced by human glioblastoma cell line U87 by targeting Fus-1 expression [18]; miR-126 regulates vascular integrity and angiogenesis, and miR-126 restoration decreases VEGF level in lung cancer cells [19], [20]; miR-130a mediates angiogenesis through downregulating antiangiogenic homeobox genes GAX and HOXA5 [21]; miR-296 level is elevated in primary brain tumor endothelial cells and regulates angiogenesis by directly targeting the hepatocyte growth factor-regulated tyrosine kinase substrate mRNA, leading to the reduction of HGS-mediated degradation of the growth factor receptors VEGFR2 and PDGFRbeta [22]. Another recent study has shown that the two RNAse III endonucleases dicer and drosha inhibit the expression of thrombospondin-1 by controlling the levels of let-7 and miR-27b, thus modulating angiogenesis [23]. However, the role of miR-21 in regulating angiogenesis remains to be elucidated. In this study, we want to study: 1) whether miR-21 regulates hypoxia inducible factor-1 (HIF-1) and vascular endothelial growth factor (VEGF) expression; 2) whether miR-21 regulates tumor angiogenesis in prostate cancer cells; 3) the signaling molecules and pathways that are regulated by miR-21 for mediating HIF-1 and VEGF expression; and 4) whether HIF-1 is the miR-21 target for regulating tumor angiogenesis.

## Materials and Methods

### Materials

Antibodies against HIF-1α and HIF-1β were from BD Biosciences (Bedford, MA, USA). Antibodies against PTEN, phospho-ERK1/2, phospho-AKT (Ser-473), and total AKT were products of Cell Signaling Technology (Beverly, MA). Antibodies against ERK2 were purchased from Santa Cruz Biotechnology (Santa Cruz, CA, USA). Pre-miR-21 and negative control precursor miRNA, anti-miR-21 inhibitor and the negative control of anti-miRNA inhibitor were purchased from Ambion (Foster City, CA). LY294002, U0126, and β-actin antibody were from Sigma (St. Louis, MO). High Capacity RNA-to cDNA Kit and Power SYBR Green PCR Master Mix were from Applied Biosystems (Carlsbad, CA).

### Cell Culture

The human prostate cancer DU145 cell was maintained in RPMI 1640 medium (Invitrogen, Carlsbad, CA, USA) supplemented with 10% fetal bovine serum (FBS), 100 units/mL penicillin, and 100 µg/mL streptomycin; and cultured at 37°C in a 5% CO_2_ incubator. Trypsin (0.25%) solution was used to detach the cells from the culture flask.

### MiRNA and Transient Transfection

To alter miR-21 levels in prostate cancer cells, DU145 cells were cultured to 50–60% confluence, and transfected with the pre-miR-21, the negative control precursor miRNA, anti-miR-21 inhibitor, and the negative control of anti-miRNA inhibitor (Ambion, Forster City, CA) using X-tremeGENE transfecting reagent (Roche Applied Science, Indianapolis, IN) in serum-free Opti-MEM medium according to the manufacturer's instruction. The final concentration of the oligomers was 25 nM. The cells were switched to fresh medium containing 10% FBS after the transfection, and cultured for 36 h. The specific protein expression was analyzed in the cells by immunoblotting, and levels of mRNA expression in the cells were analyzed by RT-PCR.

### Quantitative RT-PCR for MiR-21

Cells were transfected with pre-miR-21, miR-21 inhibitor or their control oligomers. After 48 h, total RNA was extracted. Reverse transcription reactions were performed by using mirVana qRT-PCR miRNA Detection Kit and hsa-miR-21 qRT-PCR primer set from Ambion. The 10 µL of reaction contained: 2 µL of mirVana 5× RT buffer, 1 µL of miR-21 RT primer, 0.4 µL of ArrayScript Enzyme Mix, 0.2 µL of total RNA, and 6.4 µL of H_2_O. The incubation condition was 37°C for 30 min, followed by 95°C for 10 min. The RT products were put on the ice for real-time PCR. Real-time reaction mixtures contained 12.5 µL of Master Mix, 0.5 µL of miR-21 primers, 2 µL of H_2_O, and 10 µL of RT products above. The program is 95°C for 30 min, followed by 40 cycles of 95°C for 15 s, and 60°C for 30 s. The expression of U6 was used as endogenous control for each sample. The relative gene expression was calculated by comparing cycle times for target PCR using the following equation: relative gene expression  = 2^−(ΔCtsample−ΔCtcontrol)^.

### RT-PCR

Cells were cultured in 6-well plate. Total RNAs were isolated with TRIzol reagent (Invitrogen, Carlsbad, CA) as described by the manufacturer. Two micrograms of RNA were used for cDNA synthesis using oligo(dT)_18_ as primer and M-MLV reverse transcriptase. Aliquots of cDNA were used to detect specific signals of VEGF by PCR using primers specific for VEGF, and for GAPDH (an internal control) as previously described [24]. The primers are designed using the untranslated sequences at 5′ and 3′ region as follows:

GAPDH sense primer, 5′- CCACCCATGGCAAATTCCATGGCA-3′;

GAPDH antisense primer, 5′-TCTAGACGGCAGGTCAGGTCCACC-3′;

VEGF sense primer, 5′-TCGGGCCTCCGAAACCATGA-3′;

VEGF antisense primer, 5′-CCTGGTGAGAGATCTGGTTC-3′.

VEGF and GAPDH were amplified by PCR for 30 cycles in a DNA Thermal Cycler (ABI, Foster City, CA) with each cycle at 95°C for 1 min, 59°C for 30 sec, and 72°C for 1 min. PCR products were separated on 1.2% agarose gels, stained with ethidium bromide, and visualized under UV light. The relative VEGF mRNA levels were normalized to those of GAPDH mRNA using Quality One analysis software (Bio-Rad, USA).

### Real-time RT-PCR for VEGF

Cells were treated as indicated and total RNAs were extracted. Reverse transcription reactions were performed by using High Capacity RNA-to cDNA Kit according to the manufacturer's instructions. The 100 ng of RT product was used for PCR reaction using Power SYBR Green PCR Master Mix. The reaction contained: 10 µL of 2× PCR Master Mix, 1 µL of forward primer, 1 µL of reverse primer, 1 µL of cDNA template, and 7 µL of H_2_O. The program is 95°C for 10 min, followed by 40 cycles of 95°C for 15 s, and 60°C for 30 s, and the melt curve was included. The primers of VEGF are: forward primer, 5′-CGAGGGCCTGGAGTGTG-3′, reverse primer, 5′-CCGCATAATCT GCATGGTGAT-3′. The primers of GAPDH are: forward primer, 5′-ATGGGTGTGAACCATGA GAAGTATG-3′, reverse primer: 5′-GGTGCAGGAGGCATTGCT-3′.

### Angiogenesis Assay on Chicken Chorioallantoic Membrane (CAM)

Fertilized chicken eggs were purchased from SPAFAS (Preston, CT), and incubated at 37°C with 70% humidity for 8 days. An artificial air sac was created, and a small window was cut in the shell over the artificial air sac as we described [25]. Cells transfected with pre-miR-21, pre-miRNA negative control, anti-miR-21 inhibitor, or negative control miRNA inhibitor were resuspended in serum-free medium, and mixed with equal volume of Matrigel (BD Biosciences, Bedford, MA). Aliquots (3×10^6^ cells, 30 µL) of the mixture were then applied onto the CAM of 9-day-old embryos. The area around the implanted Matrigel was photographed 4 days after the implantation, and the number of blood vessels was obtained by counting the branching of blood vessels. Assays for each treatment were carried out using 8–10 chicken embryos.

### Immunoblotting

Immnoblotting was performed as described previously [26]. In brief, cells were treated as indicated and lysed with the immune precipitation buffer (150 mM NaCl, 100 mM Tris, pH 8.0, 1% Triton X-100, 1% deoxycholic acid, 0.1% SDS, 5 mM EDTA, and 10 mM NaF) supplemented with 1 mM sodium vanadate, 0.5 mM dithiothreitol, 1 mM phenylmethylsulfonyl fluoride, 2 µM leupeptin, 2 µM aprotinin, and 2 µM pepstatin on ice for 30 min. Total cellular proteins were assayed using Bio-Rad protein assay reagent (Richmond, CA). Aliquots of the protein extracts were resolved in SDS/polyacrylamide gels, and transferred to nitrocellulose membranes. Membranes were blocked with 5% nonfat dry milk in 1× Tris-buffered saline, and incubated with antibodies against HIF-1α, HIF-1β, phospho-AKT, total AKT, phospho-ERK1/2, and total ERK2. Specific protein signals were detected by incubating with horseradish peroxidase-conjugated antibodies and a chemiluminescence reagent (Pierce Biotechnology, Rockford, IL).

### Statistical Analysis

All values in the present study were reported as Mean ± SD from three independent experiments. Two-sided student's unpaired *t* test was used for statistical analyses. *P* values less than .05 were considered statistically significant.

## Results

### MiR-21 overexpression increased HIF-1 and VEGF expression, and induced angiogenesis

**Figure 1:**
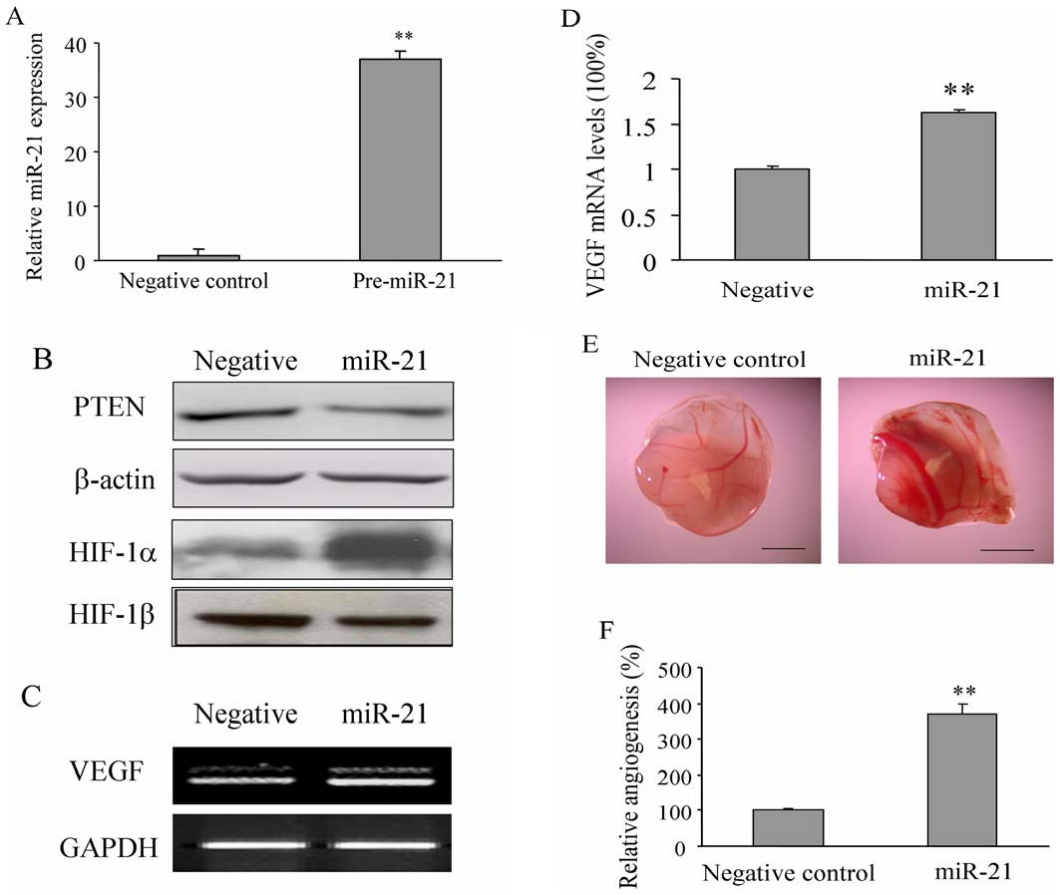
MicroRNA miR-21 overexpression increased HIF-1α and VEGF expression and induced tumor angiogenesis. (A) Human prostate cancer cells DU145 were transfected with pre-miRNA negative control or pre-miR-21 at 25 nM. After the transfection for 36 h, cells were collected and subjected to qRT-PCR for miR-21 expression. (B) DU145 cells were treated as in *A*, the total protein was extracted and subjected to Western blotting assay. (C–D) DU145 cells were collected after the transfection for 36 h. Total RNAs were extracted and analyzed for VEGF and GAPDH mRNA expression by RT-PCR analysis (C), and by real-time RT-PCR (D). (E) DU145 cells were transfected as above. After 24 h, cells (2×10^6^cells, 15 µl) were mixed with 15 µl Matrigel. The cell mixture was implanted onto the CAM of a 9-day old chicken embryo. After 4 days of implantation, the CAM was cut off, and the amount of blood vessels on the CAM induced by the DU145 plugs was determined from eight different replicated experiments. Representative plugs from negative control and miR-21 treatment groups were shown. *Scale*, 2 mm. (F) The number of blood vessels were counted from replicate experiments, and normalized to that of the negative control group as relative angiogenesis. The data are mean±SD from 6 replicates. ** indicates the significant increase when compared to the pre-miRNA negative control (*p*<0.01).

HIF-1 is a heterodimeric transcription factor, composed of two subunits: HIF-1α and HIF-1β. HIF-1α is induced by hypoxia, growth factors, and oncogenes; whereas HIF-1β protein is constitutively expressed in the cells [27], [28]. In most kinds of human cancers, HIF-1 activity is increased due to the genetic alterations or/and intratumoral hypoxia. To test whether miR-21 overexpression affects HIF-1 expression, prostate cancer cells DU145, the PTEN wild type cells, were transfected with Pre-miR-21 or negative control precursor miRNA. The expression of miR-21 increased to 37-fold after pre-miR-21 transfection when compared to the negative control, indicating that transfection of pre-miR-21 increased mature miR-21 expression (Fig. 1A). Consistent with previous study, overexpression of miR-21 suppressed PTEN expression [4]. We found that overexpression of miR-21 strongly increased expression of HIF-1α, but not HIF-1β (Fig. 1B). Vascular endothelial growth factor (VEGF) is one of the important downstream targets of HIF-1α and is a strong angiogenesis inducer [29]–[32]. To further evaluate whether the increase of miR-21 also increased VEGF expression at mRNA level, the expression of VEGF and GAPDH was tested by semi-quantitative RT-PCR and real-time RT-PCR. As shown in Figs. 1C and D, cells transfected with pre-miR-21 had 1.6-fold mRNA level of VEGF compared with cells transfected with negative control precursor miRNA. Given the crucial role of HIF-1α and VEGF in regulating tumor angiogenesis, we hypothesized that miR-21 can induce angiogenesis. To test our hypothesis, human prostate cancer cell DU145 was transfected with pre-miR-21 or pre-miRNA negative control. After the transfection for 24 h, the cells were mixed with Matrigel, and implanted onto the CAM of a 9-day old chicken embryo to perform angiogenesis assay. As we expected, the number of branches of microvessels in the pre-miR-21-transfected cells increased to 3.5-fold of the control (Figs. 1E and F). These results suggest that miR-21 induces tumor angiogenesis by upregulating the expression of HIF-1α and VEGF.

### MiR-21 inhibitor decreased miR-21-inducing HIF-1 and VEGF expression, and inhibited tumor angiogenesis

**Figure 2:**
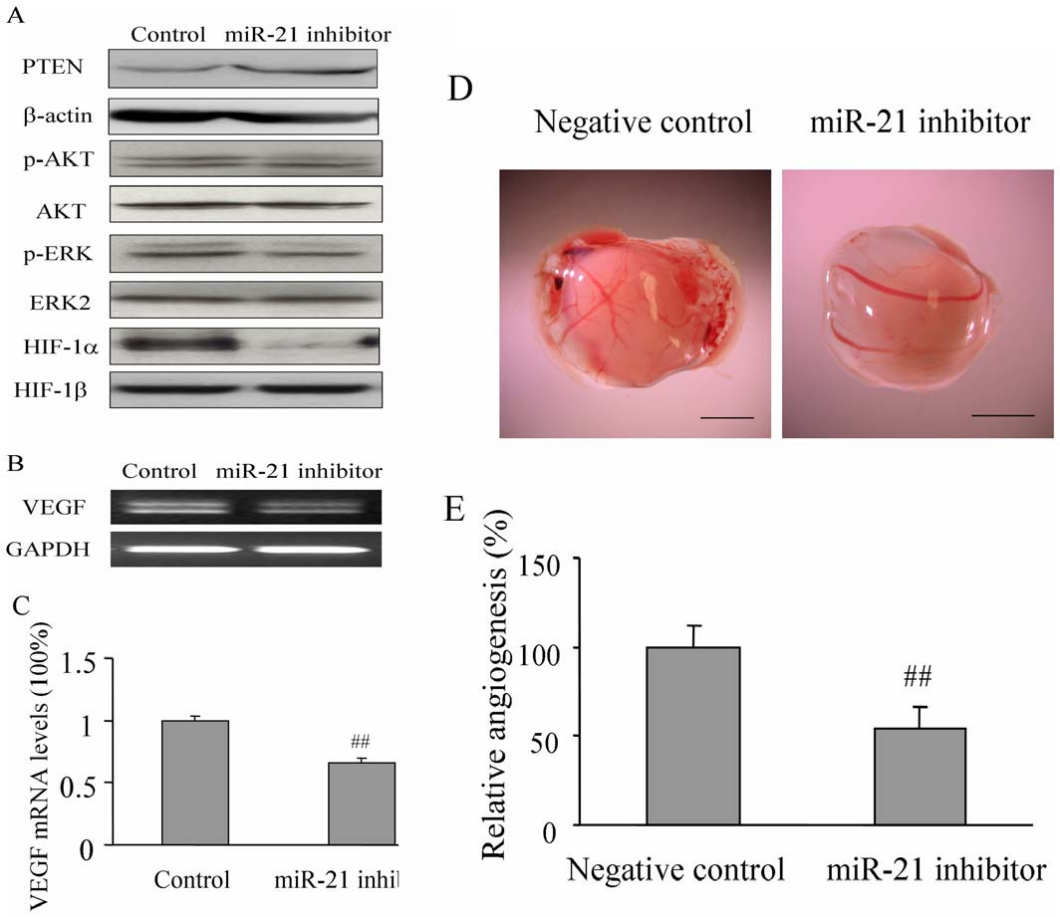
The inhibitor of miR-21 inhibited miR-21-inducing expression of HIF-1α and VEGF, activation of AKT and ERK1/2, and angiogenesis. (A) The total proteins were prepared 36 h after transfection, and analyzed by immunoblotting for the expression of PTEN, β-actin, p-AKT, AKT, p-ERK1/2, ERK2, HIF-1α, and HIF-1β. (B–C) The expression levels of VEGF in the cells described as above were analyzed by RT-PCR (B), and by real-time RT-PCR (C). (D) The cells above were used for angiogenesis assay. Representative plugs from the negative control and miR-21 inhibitor treated groups were shown. *Scale*, 2 mm. (E) The number of blood vessels were counted from replicate experiments, and normalized to that of the control group as relative angiogenesis. The data are the mean ±SD from 6 replicate experiments. ##, indicates the significant decrease when compared to that of the control (*p*<0.01).

To further test the role of miR-21 in regulating tumor angiogenesis, we overexpressed miR-21 inhibitor, which is chemically modified, single stranded nucleic acids designed to specifically bind to and inhibit endogenous miR-21 molecules. To determine whether it can inhibit tumor angiogenesis and the expression of HIF-1 and VEGF by blocking AKT and ERK1/2 pathways, DU145 cells were transfected with anti-miR-21 inhibitor and the negative control of miRNA inhibitor. When compared to the negative control of miRNA inhibitor, transfection with anti-miR-21 inhibitor increased PTEN expression, demonstrating that miR-21 inhibitor affects miR-21 direct target expression in DU145 cells (Fig. 2A). The miR-21 inhibitor treatment decreased the endogenous AKT and ERK activation, and HIF-1α but not HIF-1β expression in the cells (Fig. 2A). The anti-miR-21 inhibitor also decreased VEGF expression to 66% (Figs. 2B and C), and significantly inhibited the angiogenesis responses induced by DU145 cells (Figs. 2D and E). These results further confirm that the downregulation of miR-21 inhibits AKT and ERK activation, and HIF-1 and VEGF expression; then inhibits miR-21-inducing angiogenesis, demonstrating the vital role of miR-21 in inducing tumor angiogenesis.

### MiR-21 targeted PTEN in inhibiting tumor angiogenesis

**Figure 3:**
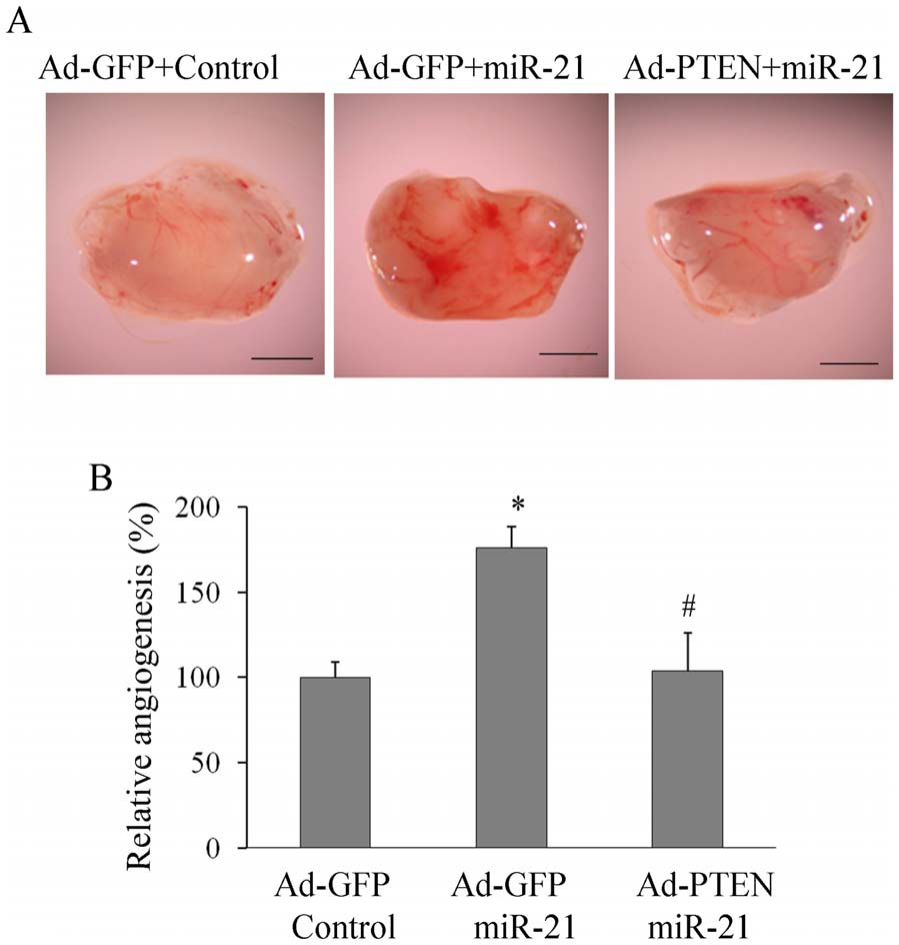
PTEN was the downstream target of miR-21 in inducing angiogenesis. (A) DU145 cells were infected with adenovirus carrying GFP (Ad-GFP) or PTEN (Ad-PTEN). After 24 h, cells were transfected with pre-miR-21 or scrambled control. The next day, cells were trypsinized and angiogenesis assay was performed as above. The representative plugs from treatments of Ad-GFP plus scrambled control, Ad-GFP plus pre-miR-21, and Ad-PTEN plus pre-miR-21 were shown in the picture. *Scale*, 2 mm. (B) The relative angiogenesis was showed in the bar graph. Results from each group are presented as mean ±SD of 6 samples. * indicates the significant increase when compared to the Ad-GFP plus scrambled control group (*p*<0.05). ^#^, indicates the significant decrease when compared to Ad-GFP plus miR-21 group (*p*<0.05).

Recent study has demonstrated that miR-21 increased cell proliferation, migration and invasion through modulating tumor-suppressor gene PTEN (4), but the role of PTEN in miR-21 inducing tumor angiogenesis remains to be elucidated. PTEN is an antagonist of phosphatidylinotidol 3-kinase (PI3K) by removing the 3′ phosphate of phosphatidylinositol 3,4,5-trisphosphate (PIP3). PTEN also inhibits the activation of mitogen-activated protein kinase (MAPK), which is the upstream regulator of ERK [33], [34]. To determine whether overexpression PTEN can inhibit miR-21 inducing prostate tumnor angiogenesis, DU145 cells were infected with adenoviruses carrying GFP or PTEN (Ad-PTEN). After 24 h, cells were transfected with pre-miR-21 or negative control precursor miRNA, and angiogenesis assay was performed as described above. Consistent with that PTEN is one of the miR-21 targets, miR-21 induced tumor angiogenesis (Ad-GFP+ negative control precursor miRNA group *versus* Ad-GFP+pre-miR-21 group: 100%±9.24% *versus* 176%±12.01%, *n* = 6); overepression of PTEN by adenovirus decreased miR-21-induced angiogenesis (Ad-GFP+pre-miR-21 group *versus* Ad-PTEN+pre-miR-21 group: 176%±12.01% *versus* 103.71%±22.1%, *n* = 6). The results suggest that PTEN mediates miR-21-induced tumor angiogenesis in CAM model (Figs. 3A and 3B).

### MiR-21 increased HIF-1 and VEGF expression through AKT and ERK1/2 pathways

**Figure 4:**
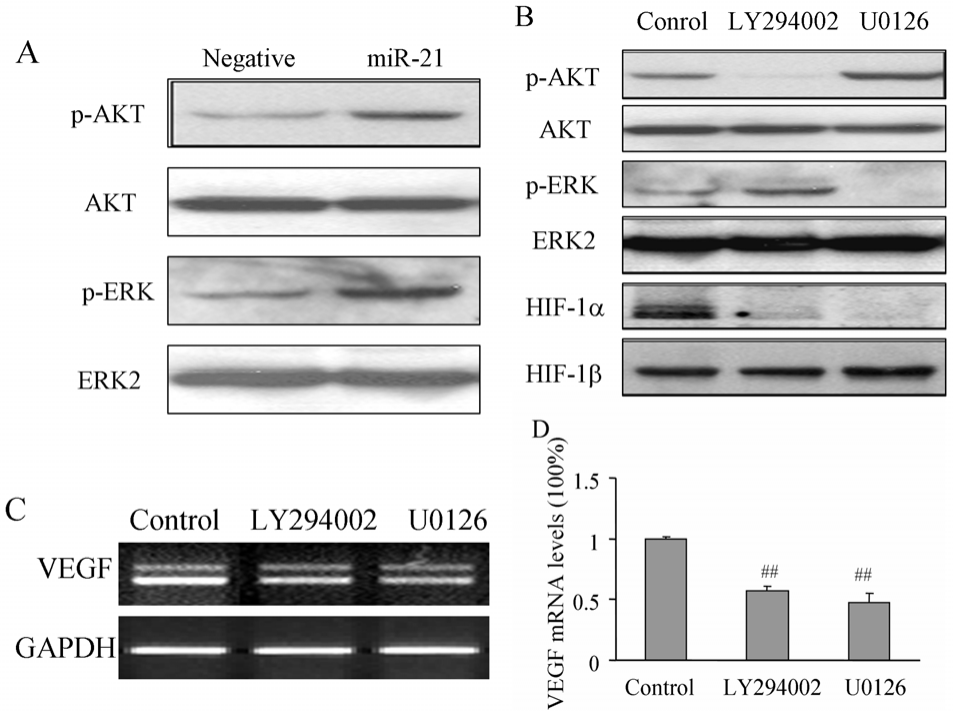
miR-21 induced the activation of AKT and ERK1/2, and the expression of HIF-1α and VEGF. (A) DU145 cells were seeded into 60 mm dishes. When cells were 60% confluence, cells were transfected with pre-miRNA negative control or pre-miR-21 as described above. Total proteins were extracted 36 h after the transfection, and analyzed for the expression of phospho-AKT (p-AKT), total AKT, phospho-ERK1/2 (p-ERK), and ERK2 by immunoblotting. (B) DU145 cells were transfected with pre-miR-21 precursor as described above. After the transfection for 36 h, the cells were treated with solvent alone, 10 µM of LY294002, or 10 µM of U0126 for 6 h. The expression levels of p-AKT, total AKT, p-ERK, ERK2, HIF-1α and HIF-1β were analyzed by immunoblotting. (C-D) After the transfection of cells with pre-miR-21, the cells were cultured for 36 h, then treated with solvent alone, 10 µM of LY294002, or 10 µM of U0126 for 12 h. VEGF mRNA expression was analyzed by RT-PCR (C), and by real-time RT-PCR (D). ##, indicates the significant decrease when compared to that of the control (*p*<0.01).

AKT (also known as protein kinase B) and extracellular signal regulated kinase (ERK) are two major signaling pathways in regulating cell proliferation, migration, and survival through its downstream targets including HIF-1α [35]-[38]. To determine the signaling molecules that are involved in miR-21-inducing HIF-1α and VEGF expression and angiogenesis, we found that overexpression of miR-21 induced AKT and ERK1/2 activation when compared to the negative control of precursor miRNA group by Western blotting (Fig. 4A). To further study the role of AKT and ERK in regulating HIF-1α and VEGF expression, DU145 cells were transfected with pre-miR-21 and treated with the inhibitors of AKT and ERK1/2, LY294002 and U0126 at 10 µM, respectively. LY294002 treatment abolished miR-21-inducing AKT activation, while U0126 inhibited miR-21-inducing ERK1/2 activation. Both LY294002 and U0126 treatment abolished miR-21-inducing HIF-1α expression (Fig. 4B). These results are consistent with previous reports that AKT and ERK are upstream regulators of HIF-1α expression. Similarly, the miR-21-inducing VEGF mRNA levels were inhibited by LY294002 and U0126 in the cells by 44% and 53%, respectively (Figs. 4C and D).

### AKT, ERK1/2, and HIF-1 are required for miR-21-inducing angiogenesis

**Figure 5:**
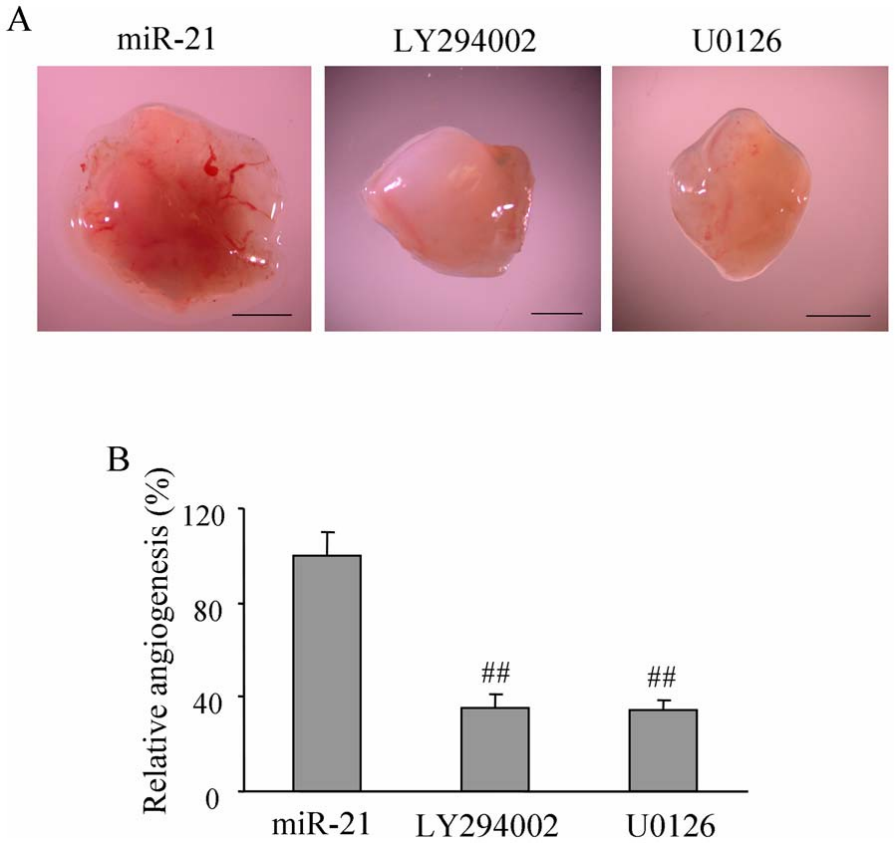
miR-21 induced angiogenesis through mediating AKT and ERK1/2 activation. (A) DU145 cells were transfected with pre-miR-21. After transfection for 36 h, the cells were trypsinized, mixed with 15 µl Matrigel in the presence or absence of 10 µM of LY294002 or U0126. Representative plugs from DMSO control, LY294002, and U0126 treatment groups were shown. *Scale,* 2 mm. (B) The number of blood vessels were counted from 8 replicate experiments, and normalized to that of the pre-miR-21 group as relative angiogenesis. ^##^, indicates the significant decrease when compared to that of pre-miR-21 treatment group (*p*<0.01).

In previous studies, we identified that miR-21 induced HIF-1 and VEGF expression through activating AKT and ERK1/2 pathways. To assess whether AKT and ERK are required for miR-21-inducing angiogenesis, DU145 cells were transfected with pre-miR-21, then treated with solvent, LY294002, or U0126 to perform the angiogenesis assay using the CAM model as before. The miR-21-inducing angiogenesis was decreased to less than 40% when the cells were treated with 10 µM of LY294002 or U0126 (Figs. 5A and B). These data suggest that both AKT and ERK1/2 are required for miR-21 in inducing angiogenesis, indicating that AKT and ERK1/2 pathways are two parallel downstream pathways of miR-21 for regulating HIF-1α and VEGF expression and angiogenesis.

**Figure 6:**
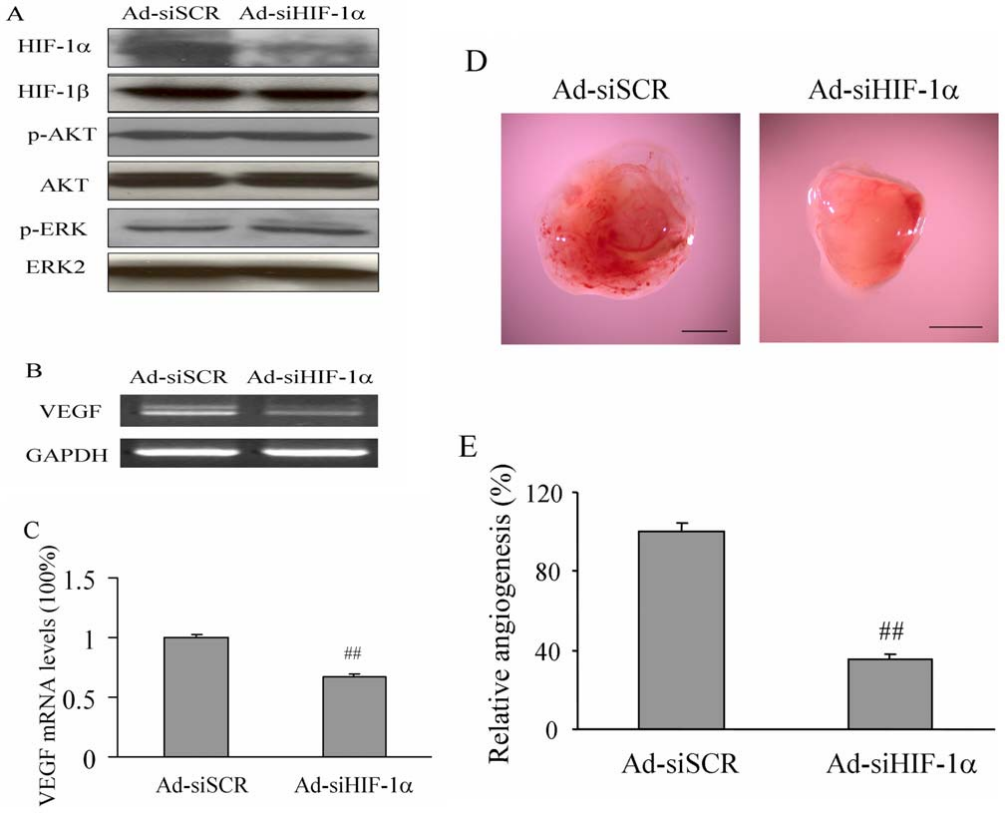
HIF-1α expression is required in miR-21 inducing VEGF expression and tumor angiogenesis. (A) DU145 cells were transfected with pre-miR-21. After 24 h incubation, cells were infected with adenovirus carrying GFP (Ad-SCR) or siRNA against HIF-1α (Ad-siHIF-1α) at 20 MOI for 24 h. The total proteins were analyzed by immunoblotting. (B–C) The expression levels of VEGF and GAPDH mRNA were analyzed by RT-PCR (B), and by real-time RT-PCR (C). (D) After DU145 cells were transfected with pre-miR-21 for 24 h, the cells were infected with Ad-GFP or Ad-siHIF-1α at 20 MOI for 24 h. Then the cells were used to perform angiogenesis assay as described above. The picture showed the representative plugs from Ad-GFP and Ad-siHIF-1α treatment groups. *Scale*, 2 mm. (E) The number of blood vessels were counted from replicate experiments, and normalized to that of the Ad-siSCR group as relative angiogenesis. The data are mean ± SD from 6 replicate experiments. ##, indicates the significant difference when compared to that of the Ad-siSCR group (*p*<0.01).

To further determine whether HIF-1α is an essential downstream molecule in mediating miR-21-inducing angiogenesis, DU145 cells were transfected with pre-miR-21 and cultured overnight. Then the cells were infected with adenovirus carrying scrambled siRNA (Ad-siSCR) or siRNA against HIF-1α (Ad-siHIF-1α). After the infection, the cells were used for the angiogenesis assay as above. Immunoblotting reslults showed that Ad-siHIF-1α infection greatly blocked HIF-1α expression when compared to the Ad-GFP treatment at protein level, but did not affect AKT and ERK1/2 activation (Fig. 6A), confirming that HIF-1α is the downstream molecule of AKT and ERK. Consistent with previous study showing that HIF-1α is the regulator of VEGF, HIF-1α siRNA also decreased miR-21-inducing VEGF mRNA expression in the cells (Fig. 6B). To further test whether the knockdown of HIF-1α is sufficient to decrease miR-21-inducing angiogenesis, the cells infected by the adenoviruses carrying scrambled siRNA and HIF-1α siRNA at 20 MOI were used for the angiogenesis assay in the CAM. As shown in Figs. 6C and D, the infection of DU145 cells by Ad-siHIF-1α decreased miR-21-inducing angiogenesis to 40% of the control group. These results suggest that upregulation of HIF-1α expression is necessary for miR-21 to induce tumor angiogenesis.

Taken together, our studies demonstrate that: 1) miR-21 up-regulated HIF-1α and VEGF expression and induced angiogenesis in prostate cancer cells; 2) miR-21 induced HIF-1α and VEGF expression through targeting PTEN, thus regulating AKT and ERK pathways; 3) both PI3K/AKT and ERK pathways are required for miR-21-inducing angiogenesis; 4) HIF-1α is an essential downstream effecter of miR-21 in mediating angiogenesis, and HIF-1α is regulated by miR-21 through the activation of AKT and ERK in the cells.

## Discussion

MicroRNAs are noncoding small RNAs that may act as oncogenes or tumor suppressor genes [39], [40]. Growing evidence shows that miR-21 overexpression is detected in various kinds of human cancers including prostate cancer, and is associated with tumor metastasis [3], [4], [41]–[46], but the direct role of miR-21 in regulating tumor angiogenesis remains to be elucidated. Angiogenesis is required for tumor growth and metastasis. VEGF plays a key role in regulating embryonic angiogenesis and tumor angiogenesis [47], [48]. HIF-1 regulates VEGF expression through transcriptional activation [29]. In this study, we demonstrated that overexpression of miR-21 in human prostate cancer cells increased the expression of HIF-1α and VEGF, and induced tumor angiogenesis. This result demonstrated that miR-21 was sufficient to induce angiogenesis through modulating the expression of HIF-1α and VEGF, providing the direct evidence that miR-21 is able to regulate tumor angiogenesis.

To further study the role of miR-21 in inducing prostate tumor angiogenesis, we used the inhibitor of miR-21, which can specifically bind to and inhibit endogenous miR-21 molecules, or its negative control oligomer, to transfect DU145 cells. The anti-miR-21 decreased 70% of endogenous miR-21 expression in the cells. In consistent with the overexpression results, inhibition of miR-21 expression in DU145 cells partially decreased AKT and ERK1/2 activation, abolished HIF-1α expression, and inhibited VEGF level to 50%. The relative angiogenesis was also inhibited 50% by the anti-miR-21, indicating that miR-21 plays an important role in mediating angiogenesis through regulating AKT and ERK activation, and HIF-1α and VEGF expression.

Next, we want to investigate the mechanism and relative signaling molecules in miR-21- inducing tumor angiogenesis. Recent study indicated that miR-21 inhibited the function of tumor suppressor PTEN expression by binding its 3′ UTR [4]. PTEN is the antagonist of phosphatidylinositol 3-kinase (PI3K), which removes the 3′ phosphate of PIP3 and attenuates signaling molecules downstream of PI3K. PTEN is frequently mutated or lost in many kinds of solid tumors [49]. PTEN is the upstream regulator of both PI3K/AKT and MEK/ERK signaling pathways. The PI3K/AKT and ERK signaling pathways play crucial roles in various intracellular cascade events including tumor angiogenesis and tumor growth [50], [51]. In accord with previous study, PTEN overexpression notably blocked miR-21-induced tumor angiogenesis, confirming that miR-21 exhibits its role partly by inhibiting PTEN expression and the recovery of PTEN expression restored its function of tumor suppressor. We also found that overexpression of miR-21 led to the activation of AKT and ERK1/2. Treatment of the cells with PI3K or ERK1/2 inhibitor LY294002 or U0126, respectively, prohibited miR-21-inducing HIF-1α and VEGF expression and angiogenesis, suggesting that both PI3K and ERK are required for miR-21-inducing angiogenesis through the downregulation of HIF-1α and VEGF expression. Taken together, miR-21 inhibited PTEN expression, which in turn decreased AKT and ERK activation for inhibiting HIF-1α and VEGF expression, thus inducing tumor angiogenesis.

HIF-1α regulates many target genes including VEGF by binding to their promoters [27]. Finally, given the important role of HIF-1 and VEGF in mediating tumor growth and angiogenesis, we hypothesize that HIF-1 is a downstream target required for miR-21-inducing angiogenesis. To test this postulation, we found that HIF-1α siRNA significantly inhibited the expression of HIF-1α and VEGF and tumor angiogenesis without the change of phospho-AKT and phospho-ERK expression. This data suggests that HIF-1α is a key downstream target of miR-21, which is required for inducing angiogenesis, and is downstream of both AKT and ERK pathways in regulating miR-21-inducing angiogenesis. Taken together, our results demonstrated that miR-21 regulates tumor angiogenesis through inducing HIF-1α and VEGF expression; activates both AKT and ERK pathways for mediating angiogenesis. HIF-1α is an important downstream target of miR-21, and is regulated by both AKT and ERK during miR-21-inducing angiogenesis. This study shows a direct role and novel mechanism of miR-21 in inducing angiogenesis and the information may be useful to develop a new anti-angiogenesis therapy for prostate cancer treatment in the future.
